# Can infrascanner be useful in hospital emergency departments for diagnosing minor head injury in children?

**DOI:** 10.34763/devperiodmed.20172101.5159

**Published:** 2017-05-29

**Authors:** Dorota Lewartowska-Nyga, Kamil Nyga, Grażyna Skotnicka-Klonowicz

**Affiliations:** 1 Oddział Kardiologii i Reumatologii dla Dzieci II Katedra Pediatrii UM w Łodzi Ośrodek Pediatryczny im. M. Konopnickiej, Centralny Szpital Kliniczny UM w Łodzi Poland; 2 Centrum Medycyny Rodzinnej i Społeczności Lokalnych UM w Łodzi Poland; 3 Oddział Kliniczny Medycyny Ratunkowej dla Dzieci II Katedra Pediatrii UM w Łodzi Ośrodek Pediatryczny im. M. Konopnickiej, Centralny Szpital Kliniczny UM w Łodzi Poland

**Keywords:** lekkie urazy głowy u dzieci, Infraskanner, tomografia komputerowa głowy, krwawienie wewnątrzczaszkowe, minor head trauma in children, Infrascanner, computed tomography, intracranial haemorrhage

## Abstract

**Aim:**

The aim of the study was to determine whether Infrascanner screening is a test which would facilitate excluding acute intracranial bleeding in children after minor head injury and thus make it possible to limit indications for computed tomography in those children.

**Material and methods:**

The study enrolled 155 children aged 2-18 years after a minor or moderate head injury. The children were assessed using the Glasgow Coma Scale, examined by Infrascanner screening. Those who had relvant indications also had head computed tomography.

**Results:**

A negative Infrascanner screening result (no intracranial bleeding) was noted in 151 children. The Infrascanner result was positive in 4 children. Head computed tomography was performed in 28 of the 155 children. The conformity of the Infrascanner result with the computed tomography image was found in 26 children: no evidence of intracranial bleeding in 24 children and confirmation of intracranial haematoma in 2 children. The sensitivity of the screening was 66.67% and its specificity 98.68%. The positive and negative predictive values of the screening were 50% and 99.34%, respectively. The reliability of the test results was 98.06%.

**Conclusion:**

The Infrascanner seems to be a useful device in diagnosing children after minor head injury in the emergency department and its portability makes it possible to use it in practically all settings. Introducing the device into management standards in children after minor head injury might facilitate selecting those after minor head injury who are not at risk of intracranial bleeding and contribute to a reduction in the number of imaging investigations being performed and decrease the number of hospitalisations.

## Streszczenie

**Materiał i metody:**

Do badania zakwalifikowano 155 dzieci w wieku 2-18 lat, po lekkim lub średnio ciężkim urazie głowy. Dzieci te były ocenione wg skali Glasgow oraz badane przy użyciu Infrascannera. Pacjenci, którzy mieli wskazania do wykonania badań obrazowych, mieli również wykonaną tomografię komputerową głowy.

**Wyniki:**

Ujemny wynik badania Infraskannerem (brak cech krwawienia wewnątrzczaszkowego) stwierdzono u 151 dzieci. U 4 dzieci wynik Infraskannera był dodatni. U 28 spośród 155 dzieci wykonano tomografię komputerową głowy. Zgodność wyniku badania Infraskannerem z obrazem tomografii komputerowej zaobserwowano u 26 dzieci: u 24 dzieci brak było cech krwawienia wewnątrzczaszkowego oraz u 2 dzieci potwierdzenie obecności krwiaka wewnątrzczaszkowego. Ponadto, odnotowaliśmy 2 wyniki fałszywie dodatnie oraz 1 wynik fałszywie ujemny. Czułość badania wyniosła 66,67%, a swoistość 98,68%. Natomiast wartość predykcyjna dodatnia badania wyniosła 50%, a wartość predykcyjna ujemna 99,34%. Wiarygodność wyników badania wyniosła 98,06%.

**Wnioski:**

Infraskanner wydaje się być przydatnym urządzeniem w diagnostyce dzieci po lekkim urazie głowy w szpitalnym oddziale ratunkowym, a jego mobilność sprawia, że może być wykorzystywany praktycznie w każdych warunkach.

Wdrożenie tego urządzenia do standardów postępowania po lekkim urazie głowy u dzieci mogłoby ułatwić wyłonienie tych pacjentów po lekkim urazie głowy, którzy nie są zagrożeni krwawieniem wewnątrzczaszkowym i korzystnie wpłynąć na zmniejszenie liczby wykonywania badań obrazowych – tomografia komputerowa głowy oraz na zmniejszenie liczby hospitalizacji.

## Wstęp

Urazy głowy są jedną z częstszych przyczyn, z powodu których dzieci trafiają do Szpitalnego Oddziału Ratunkowego (SOR). Dane epidemiologiczne wskazują na wagę tego zjawiska w skali światowej. W Stanach Zjednoczonych na skutek urazu głowy rocznie umiera około 7400 dzieci, a 29 000 doznaje trwałej niepełnosprawności. Do SOR-u trafia około 500-650 000 niepełnoletnich z czego 50-95 000 wymaga hospitalizacji [1, 2, 3, 4, 5, 6, 7]. Podobną sytuację odnotowuje się w wielu krajach europejskich [3, 4]. W 80% przypadków urazy głowy u dzieci to tzw. lekkie urazy głowy (LUG) niepowodujące zazwyczaj poważnych uszkodzeń mózgu, jednak nie pozbawione są powikłań [8, 9].Wg różnych źródeł ryzyko powikłań po LUG obserwuje się w 1,2-7% przypadków [1, 2, 3, 10]. Postępowanie z dzieckiem po LUG z uwagi na brak jednoznacznej definicji oraz jednolitych standardów postepowania stanowi problem diagnostyczno-terapeutyczny. Jak zauważa Strzyżewski i wsp. „Współcześnie istnieje spore zamieszanie w terminologii lekkich urazów głowy” [11]. W wielu ośrodkach w USA badanie przy użyciu tomografii komputerowej (TK) głowy jest wykonywane w każdym przypadku LUG [2, 12], chociaż Amerykańska Akademia Pediatrii (AAP) uważa, że u dziecka po LUG bez utraty przytomności, takie postępowanie jest niezasadne (ryzyko nowotworów − zbyt duże dawki napromieniowania, jak i zbyt wysokie koszty) [2, 12, 13, 14]. Ustalenie zatem sposobu postepowania z dzieckiem po LUG ma istotne znaczenie dla ochrony dziecka przed nadmierną diagnostyką z wykorzystaniem promieniowania jonizującego oraz zmniejszenia ryzyka powikłań związanych z koniecznością znieczulenia ogólnego do wykonania TK u najmłodszych dzieci [7, 13, 15, 16, 17].

Ostatnio pojawiły się informacje o przydatności w diagnostyce urazów głowy przenośnego i prostego w użyciu urządzenia wykorzystującego promieniowanie podczerwone − Infraskannera (ryc. 1), wykrywającego krwawienia wewnątrzczaszkowe o objętości większej niż 3,5 cm^3^ zlokalizowane na głębokości do 2,5 cm od powierzchni mózgu, lub 3,5 cm od powierzchni czaszki. Istotą działania urządzenia jest ocena różnicy gęstości optycznej (=OD) między prawą, a lewą półkulą mózgu, która w warunkach prawidłowych jest taka sama [5, 18, 19, 20, 21, 22].

**Ryc. 1:**
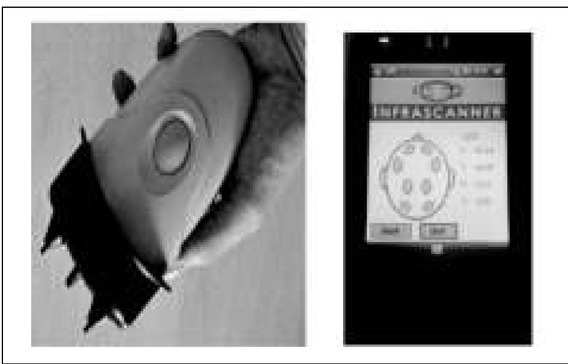
Infrascanner (czujnik i palmtop) − wynik negatywny badania [materiał własny]. Fig. 1. Infrascanner (sensor and palmtop) – negative result of screening [own material].

**Tabela I:** Kryteria wyłączenia z badania. Table I. Criteria for exclusion from the study.

Kryteria wyłączenia: *Exclusion criteria*:
Uraz głowy >3 dób (>72 h) *Head injury > days (>72 hours)*
Dzieci <2 roku życia *Children <2 years of age*
Dzieci z przeszłością neurologiczną *Children with neurological history*
Uraz głowy doznany w mechanizmie zasłabnięcia albo omdlenia wazowagalnego *Head injury sustained in fainting or vasovagal episode mechanism*
W miejscu przewidzianym do badania rozległe rany, otarcia labo krwiaki uniemożliwiające wykonanie badania *Extensive wounds, abrasions or haematomas in the site to be examined*

## Cel pracy

Ustalenie, czy badanie przy użyciu Infraskannera ułatwiłoby wykluczenie u dzieci po lekkim urazie głowy, ostrego krwawienia wewnątrzczaszkowego i tym samym pozwoliłoby ograniczyć liczbę wskazań do tomografii komputerowej.

## Materiał i metody

Do badania przeprowadzonego w okresie od jesieni 2011 roku do wiosny 2016 roku, zakwalifikowano 155 dzieci po lekkich i średniociężkich urazach głowy, które zgłosiły się do SOR-u dla dzieci i których rodzice wyrazili zgodę na przeprowadzenie badania. Kryteria wykluczenia z badania przedstawiono w tabeli I. W oparciu o skalę Glasgow (GCS), dzieci przydzielono do dwóch grup: lekkich urazów głowy (GCS 15-13 pkt) i średnio ciężkich urazów głowy (GCS 12-8 pkt). Dzieci poddane były ocenie chirurgicznej, neurologicznej oraz w wybranych przypadkach okulistycznej i laryngologicznej. Wszystkie dzieci badane były Infraskannerem (przez osoby odpowiednio wcześniej przeszkolone). Wynik badania przedstawiony był w postaci graficznej. Kolor zielony (=OD ≤0,2) w obrębie ww. ośmiu punktów badania − oznaczał brak krwawienia wewnątrzczaszkowego. Kolor czerwony (∆OD >0,2), w którymkolwiek z obszarów badania, sugerował obecność krwawienia wewnątrzczaszkowego. Celem eliminacji błędu, wg zaleceń producenta badanie powtarzano dwukrotnie. Trzykrotny wynik pozytywny w danej okolicy stanowił wskazanie do pogłębienia diagnostyki o TK głowy. Ponadto u dzieci, u których były wskazania wykonano TK głowy (lekarz opisujący nie wiedział o badaniu Infraskannerem). W grupie dzieci z ujemnym wynikiem Infraskannera potwierdzono ten wynik na drodze kontaktu z rodzicami dziecka po upływie 2-3 miesięcy. Uzyskane wyniki badania poddano analizie statystycznej dla cech mierzalnych (ilościowych) i dla cech niemierzalnych (jakościowych). Dla scharakteryzowania cech ilościowych obliczono średnią arytmetyczną

(x) i medianę (Me). Za miarę rozrzutu przyjęto odchylenie standardowe (SD). W analizie statystycznej dla cech niemierzalnych (jakościowych) zastosowano Test McNemara-służący do analizy zmiennych jakościowych związanych. A ponadto oceniono czułość, swoistość oraz wartość predykcyjną uzyskanych wyników (tab. II). Na przeprowadzenie badania uzyskano zgodę Komisji Bioetycznej o numerze RNN/50/12/KB.

**Tabela II:** Ocena czułości i swoistości. Table II. Evaluation of sensitivity and specificity.

		Wynik badania CT *CT results*			
		dodatni (+*) positive (+)*	Ujemny (-) *negative (-)*	Suma *Sum*	
Wynik badania Infraskanerem *Infrascanner screening result*	dodatni (+) *positive (+)*	prawdziwie dodatni (PD) *true positive (TP)*	fałszywie dodatni (FD) *false positive (FP)*	PD+FD *TP+FP*	wartość predykcyjna dodatnia *positive predictive value*
	ujemny (-) *negative (-)*	fałszywie ujemny (FU) *false negative (FN)*	prawdziwie ujemny (PU) *true negative (TN)*	FU+PU *FN+TN*	wartość predykcyjna ujemna *negative predictive value*
	Suma	PD+FU TP+FN	FD+PU FP+TN	PD+FD+FU+PU TP+FP+FN+TN	
	*Sum*	Czułość *Sensitivity*	Swoistość *Specificity*		

## Wstępne wyniki

Spośród 220 dzieci, które po lekkim lub średniociężkim urazie głowy trafiły do SORu Ośrodka Pediatrycznego Centralnego Szpitala Klinicznego w Łodzi i spełniły kryteria włączania. Do badania zakwalifikowano 155 dzieci (ryc. 2). Charakterystykę grupy badanej przedstawiono w tabeli III. Przyczyny urazu oraz objawy zgłaszane przez dzieci przedstawiono w tabelach IV i V.

**Ryc. 2:**
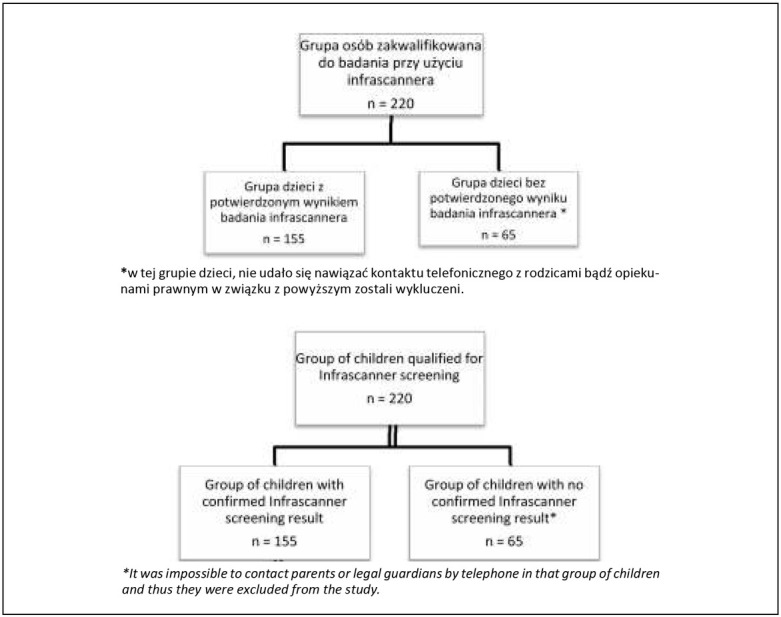
Diagram włączenie pacjentów do badania. Fig. 2. Flow chart of patient inclusion in the study.

**Tabela III:** Charakterystyka grupy badanej. Table III. The characteristics of the study group.

Cechy	Liczba pacjentów N=155
*Criteria*	*Number of patients N=155*
	2-18 lat
	*2-18 year*s
Wiek dzieci	Średnia wieku 12,2 lat
*Children ag*e	*Mean age of 12.2 years*
	Mediana 13 lat
	*Median 13 years*

	Dziewczynki 58 (37,4 %)
Płeć	*Girls*
*Sex*	Chłopcy 97 (62,6%)
	*Boys*

Ocena w skali GCS:	
*Raiting to the GCS*:	
− 15 pkt *(15 points)*	150 (96,7%)
− 14 pkt *(14 points)*	3
− 14-13 pkt *(14-13 points)*	1
− 12 pkt *(12 points)*	1

Badanie fizykalne:	
Physical examination:	
− Bez odchyleń od stanu prawidłowego *No abnormalities*	90 (58,1%)
− Krwiak w obrębie powłok skórnych twarzoczaszki oraz czaszki*	32 (20,6%)
Haematomas was found within the integument of the face and skull*	
− Rany lub otarcia naskórka * *Wounds or skin abrasion*s	25 (16,1%)
− Obrzęk, zasinienie powłok tylko w okolicy twarzoczaszki *Oedema and bruising of the integument solely in the facial region*	8 (5,2 %)

Ocena neurologiczna:	
*The neurological condition*: prawidłowa	
*No abnormalities*	152 (98%)
−odchylenia od normy:	
*Abnormalities*:	3
− zaburzenie czucia	
*sensory abnormalities*	1
− zaburzenia świadomości, bez objawów ogniskowych, wstępnie ocenionego dziecka w GCS na 12 pkt *Consciousness disturbances with no focal signs in 1 child preliminar*ily assessed at *12 on the GCS*	1
− dziecko ocenione w GCS na 14-13 pkt *Child assessed at 14-13 on the GCS*	1

Ocena okulistyczna:	
*The ophthalmological condition*: Prawidłowa	44 (28%)
*No abnormalities*	
Odchylenia od normy:	41
*Abnormalities*	
− krwiaki powiek	
*Haematomas of the eyelids*	3

Ocena laryngologiczna: The laryngological condition:	2
Prawidłowa *No abnormalities*	2

* Lokalizacja umożliwiła przeprowadzenie badania Infraskannerem. *Their location allowed for Infrascanner screening.

**Tabela IV:** Przyczyna urazu w grupie badanej. Table IV. Cause of injury in the study group.

Przyczyna urazu *Cause of injury*	Liczba pacjentów N=155 *Number of patients n=155*	
Upadek z wysokości *Fall from a height*	28	18,1%
Wypadek komunikacyjny *Traffic accident*	28	18,1%
Upadek jednopoziomowy *Same-level fall*	36	23,2%
Bezkolizyjny wypadek *Non-collision accident*	12	7,7%
Pobicie *Battery*	15	9,7%
Zespół dziecka maltretowanego *Battery child syndrome*	0	0
Uderzenie o przedmiot nieruchomy *Hitting on an unmoving object*	16	10,3%
Uderzenie o przedmiot będący w ruchu *Hitting on a moving object*	18	11,6%
Inne *Other*	2	1,3%

**Tabela V:** Objawy w grupie badanej. Table V. Symptoms and signs in the study group.

Objawy *Symptoms and signs*	Liczba pacjentów N= 155 *Number of patients N=155*	
Utrata przytomności *Loss of consciousness*	46	19,4%
Wymioty *Vomiting*	44	18, 6%
Ból głowy *Headache*	91	38,4%
Niepamięć wsteczna *Retrograde amnesia*	25	10,5%
Drgawki *Convulsions*	0	0
Objawy ogniskowe *Focal signs*	2	0,8%
Bez dolegliwości *No symptoms*	29	12,2%

U 13 spośród 155 badanych (8,4%) lekarz dyżurny zlecił wykonanie badania radiologicznego czaszki, które nie wykazało obecności szczeliny złamania.

Badanie za pomocą Infraskanera wykonano u wszystkich 155 dzieci po LUG zgodnie z zaleceniami producenta w okresie od ½ godziny do 72 godzin po urazie (mediana do 24 godzin). Brak cech krwawienia wewnątrzczaszkowego, czyli ujemny wynik badania (=OD ≤0,2), stwierdzono u 151 spośród 155 badanych (97,4 %) (ryc. 1). U wszystkich dzieci z ujemnym wynikiem badania Infraskannerem wynik ten potwierdzono telefonicznie w okresie 2-3 miesięcy od urazu.

Podejrzenie krwawienia wewnątrzczaszkowego, czyli dodatni wynik badania (=OD >0,2), odnotowano u 4 spośród 155 pacjentów (2,6%). W 2 przypadkach tj.: u 11-letniej dziewczynki, która doznała urazu głowy w wypadku samochodowym, z utratą przytomności, z niepamięcią wsteczną, wymiotującą, ocenioną w skali GCS na 12 punktów (splątana, lokalizowała ból, otwierała oczy na bodziec słowny) i u 3-letniej dziewczynki, która doznała lekkiego urazu głowy oraz złamała lewy obojczyk wskutek upadku ze schodów, ocenionej w skali Glasgow na 15 punktów, u której po 72 godz. od zadziałania urazu pojawił się obrzęk w lewej okolicy ciemieniowej − dodatni wynik badania Infraskannerem został potwierdzony w TK głowy, która w obu przypadkach wykazała obecność krwiaka wewnątrzczaszkowego (ryc. 3). W trzecim przypadku u dziewczynki 16-letniej, po wypadku samochodowym, ocenionej w skali GCS na 14 pkt, z niepamięcią wsteczną, bólami głowy oraz rozległym krwiakiem w okolicy skroniowej lewej, w TK głowy stwierdzono jedynie obecność krwiaka podczepcowego (ryc. 4). W czwartym przypadku ze względu na dobry stan ogólny dziecka i brak objawów neurologicznych odstąpiono od badania TK głowy. Słuszność tej decyzji potwierdzono telefonicznie po upływie około 2-3 miesięcy. U 28 dzieci z grupy badanej (18,1%) niezależnie od badania Infraskannerem wykonano TK głowy. Wskazaniem do TK była obecność objawów klinicznych (utrata przytomności, niepamięć wsteczna, wymioty po urazie oraz utrzymujące się bóle głowy) lub wysokoenergetyczny mechanizm urazu. Zgodność wyniku badania Infraskannerem z obrazem TK zaobserwowano u 26 dzieci (92,9%) − potwierdzenie braku cech krwawienia wewnątrzczaszkowego u 24 dzieci i potwierdzenie obecności krwiaka wewnątrzczaszkowego u 2 dzieci.

**Ryc. 3:**
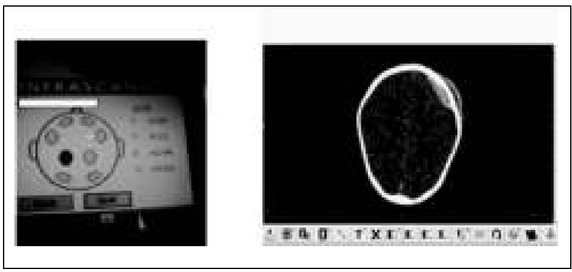
Pozytywny wynik Infraskannera. W TK głowy w okolicy ciemieniowo-czołowej lewej krwiak nadtwardówkowy podostry z cechami niewielkiej progresji (materiał własny). Fig. 3. ositive Infrascanner screening result. Head CT revealed subacute epidural haematoma with slight progression evidence in the left parietofrontal region (own material).

**Ryc. 4:**
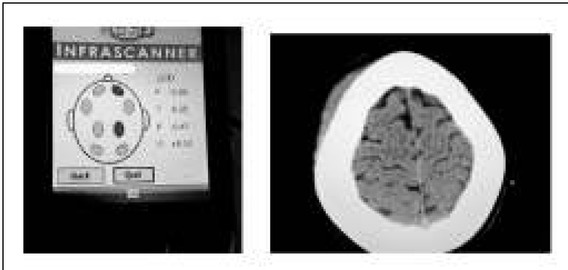
Wynik fałszywie dodatni. Infrasacnner wynik badania dodatni, TK-bez cech krwawienia wewnątrzczaszkowego, jedynie krwiak podczepcowy (materiał własny). Fig. 4. False positive result. Positive Infrascanner screening result. Head CT with no evidence of intracranial bleeding but only subgaleal haematoma (own material).

Brak zgodności wyniku badania Infraskannerem z obrazem TK głowy stwierdzono u 2 dzieci. W jednym przypadku otrzymano fałszywie ujemny wynik badania Infraskannerem. Infraskanner nie wykazał bowiem obecności 2 mm krwiaka przymózgowego w prawej okolicy czołowej, którego obecność wykazano w obrazie TK. Brak tej zgodności wynikał prawdopodobnie z wielkości krwiaka. W drugim przypadku dziecka z dużym krwiakiem w tkankach miękkich uzyskano fałszywie dodatni wynik Infraskannerem. TK głowy wykazała u tego dziecka tylko krwiak podczepcowy. Zgodność badania Infraskannerem ze stanem klinicznym dziecka po urazie albo z wynikiem TK stwierdzono u 152 osób spośród 155 przypadków (98,1%). W jednym przypadku odnotowano fałszywie ujemny wynik badania oraz w dwóch przypadkach fałszywie dodatni wynik badania.

W badanej grupie pacjentów czułość badania została oceniona na 66,67% co oznaczało, że 66,67% dzieci z dodatnim wynikiem badania Infraskannerem zagrożonych było krwiakiem wewnątrzczaszkowym. Swoistość badania wyniosła 98,68%, co pozwalało uznać, że wśród badanych u 98,68% Infraskanner może wykluczyć krwawienie wewnątrzczaszkowe. Wartość predykcyjna dodatnia badania wyniosła 50%, a wartość predykcyjna ujemna na 99,34%. Wiarygodność wyniku badania wyniosła 98,06%. Testem McNemara wykazano, iż rozbieżność w wynikach fałszywie ujemnych pomiędzy Infraskannerem, a TK głowy jest nieistotna statystycznie (x2=0,5 p>0,05).

## Dyskusja

Poszukiwanie bezpiecznego sposobu postępowania diagnostyczno-terapeutycznego dla dziecka po LUG ma szczególne znaczenie zwłaszcza dla szpitalnych oddziałów ratunkowych, ponieważ to tutaj najczęściej zgłasza się dziecko po urazie głowy i tutaj podejmowane są decyzje co do dalszego postępowania z dzieckiem. Decyzje te muszą uwzględniać przede wszystkim dobro pacjenta tzn. jego bezpieczeństwo i zapobieganie powikłaniom po urazie, ale również ochronę dziecka przed zjawiskiem nadmiernej diagnostyki (narażenie na promieniowanie jonizujące) i niepotrzebną hospitalizacją oraz racjonalizację kosztów leczenia. Działania takie podejmowane sąw licznych ośrodkach krajowych i zagranicznych [2, 3, 8, 16, 17, 23].

Pojawiające się pojedyncze doniesienia o przydatności badania Infraskannerem (urządzenia wykorzystującego promieniowanie podczerwone) w diagnostyce pacjentów po urazie głowy jako badania przesiewowego, ułatwiającego kwalifikuję pacjenta do badania TK wpisują się w te działania i sugerują rozpowszechnienie tych badań. Wysoka czułość badania Infraskannerem stwierdzana u pacjentów z krwawieniem wewnątrzczaszkowym zainspirowała do podjęcia tego badania u dzieci z LUG w warunkach szpitalnego oddziału ratunkowego [5, 9, 16, 18, 19, 20, 21, 22].

Infraskanner jest urządzeniem wykorzystywanym w medycynie od niedawna i dotychczas opublikowano zaledwie kilkanaście prac u dorosłych i pojedyncze prace u dzieci oceniające praktyczność i skuteczność tego urządzenia, w wykrywaniu krwawień wewnątrzczaszkowych po urazach głowy [5, 9, 16, 18, 19, 20, 21, 22]. Robertson i wsp. przeprowadzili badanie na dużej grupie pacjentów 365 osób w wieku od 1 do 89 r.ż., Leon-Carrion i wsp. na grupie 35 pacjentów w wieku 17-76 lat. Salonia i wsp. u dzieci w wieku 0-14 lat, Bressan i wsp. badali dzieci do 15 r.ż. po urazach głowy z grupy średniego i wysokiego ryzyka, natomiast Semanova i wsp. w grupie 7 m-cy -17 lat w grupach badawczych nie przekraczających 110 dzieci [5, 18, 19, 20, 21, 22]. Salonia i wsp. dokonywali podziału pacjentów na podstawie wyniku TK głowy (prawidłowy oraz nieprawidłowy) porównując uzyskany wynik badania z wynikiem Infraskannera. Natomiast Semenova i wsp. podobnie jak w pracy własnej badali dzieci po LUG dokonując podziału z uwzględnieniem wskazań do TK głowy.

Badaniem własnym objęto grupę 155 dzieci w wieku od 2 do 18 roku życia. Badani, to dzieci głównie po LUG, co różni badaną grupę od grup opisywanych przez innych autorów. W pracy własnej oceniano bowiem przydatność tego badania u dzieci z lekkimi urazami głowy celem ograniczenia w tej grupie badań obrazowych (RTG, TK). Bressan i wsp. nie badali w ogóle dzieci z urazem niskiego ryzyka lub z tzw. banalnym urazem głowy oraz wykluczyli dzieci, które miały już wykonane badania obrazowe głowy w innym ośrodku. Podobnie, jak w pracy własnej autorzy ci potwierdzili prawdziwość ujemnego wyniku badania Infraskannerem również na drodze kontaktu telefonicznego z rodzicami po upływie kilku miesięcy od urazu [18]. W grupie badanej dzieci po LUG przeprowadzone badanie Infraskannerem wykazało czułość 66,67% oraz swoistość 98,68%. Robertson i wsp. w grupie pacjentów z krwawieniem wewnątrzczaszkowym (nie zależnie od wielkości i typu krwawienia) uzyskali podobnie wyniki, czułość badania w 68,7%, natomiast swoistość nieco mniejszą, bo 90,7%. Ponadto w grupie pacjentów z krwawieniem wewnątrzczaszkowym, u których krwiak miał rozmiary na tyle istotne, że dawał kliniczne objawy, obserwowano czułość rzędu 88%, a swoistość 90,7% [21]. Bressan i wsp., wykazali swoistość badania na 93%, a wartość predykcyjną ujemną na 100% w wykrywaniu krwawień wewnątrzczaszkowych [18]. Salonia i wsp. uzyskali czułość rzędu 100% a swoistość 80% w wykrywaniu krwawienia wewnątrzczaszkowego [5]. Podobne wyniki wykazali Semenova i wsp., którzy dowiedli w wykrywaniu krwawień wewnątrzczaszkowych u dzieci czułość i swoistość odpowiednio na 100% i 91%[22].

Uzyskane wyniki w pracy własnej wykazały wysoką swoistość 98,68% oraz wysoką ujemną wartość predykcyjną 99,34%, co również potwierdzają obserwacje innych autorów [5, 18, 21, 22]. Pozwala to przypuszczać, iż u dzieci z ujemnym wynikiem badania Infraskannerem zagrożenie krwawieniem wewnątrzczaszkowym jest znikome (0,6%) co potwierdza również test wiarygodności 98,06%. W świetle tych danych rodzi się pytanie, czy u dzieci po lekkich urazach głowy po uwzględnieniu kryterium wykluczenia, nie należy diagnostyki rozpoczynać od wstępnego badania Infrskannerem zwłaszcza, że obserwacje własne oraz dane z aktualnie dostępnej literatury wskazują na brak działań niepożądanych wykorzystanego urządzenia [5, 9, 18, 19, 20, 21, 22]. Jednocześnie zwracają uwagę pewne ograniczenia tj. rozległe otarcia, rany, krwiaki podskórne w miejscach przewidzianych do badania, które mogą być powodem wyniku fałszywie dodatniego, stąd w tych przypadkach nie zaleca się wykonywania tego badania. Warto pamiętać o innych czynnikach tj. gęstość włosów, fryzura czy też przymusowe ułożenie pacjenta, które mogą wpłynąć na wydłużenie czasu badania. Niezależnie jednak od ww. ograniczeń, badanie Infarsaknnerem może stanowić istotny przełom w diagnostyce dzieci po LUG.

## Wnioski

Uzyskane wyniki badania (wysoka swoistości 98,68% i wartość predykcyjna ujemna 99,34%) wskazują, że u dzieci po LUG uzyskanie ujemnego wyniku badania Infraskannerem, może być przydatne do podjęcia decyzji o odstąpienia od badania tomografii komputerowej głowy.

### Podziękowania

Podziękowania dla całego zespołu Szpitalnego Oddziału Ratunkowego Ośrodka Pediatrycznego im. M. Konopnickiej, Centralnego Szpitala Klinicznego UM w Łodzi.
